# Training Characteristics, Performance, and Body Composition of Three U23 Elite Female Triathletes throughout a Season

**DOI:** 10.3390/sports12020053

**Published:** 2024-02-07

**Authors:** Sergio Sellés-Pérez, Hector Arévalo-Chico, José Fernández-Sáez, Roberto Cejuela

**Affiliations:** 1 Physical Education and Sports, Faculty of Education, University of Alicante, 03690 San Vicente del Raspeig, Spain; hector.arevalochico@ua.es (H.A.-C.); roberto.cejuela@ua.es (R.C.); 2 Unitat de Suport a la Recerca Terres de l’Ebre, Fundació Institut, Universitari per a la Recerca a l’atenció Primària de Salur Jordi Gol i Urina (IDIAPGol), 43500 Tortosa, Spain; jfernandez@idiapjgol.info; 3 Facultat de Enfermería, Campus Terres de l’Ebre, Universitat Rovira i Virgili, 43500 Tortosa, Spain

**Keywords:** endurance training, training intensity distribution, training load, training periodization, triathlon, physiological variables

## Abstract

(1) Background: There is a lack of data on the long-term training characteristics and performance markers of elite young female endurance athletes. The aim of this study was to present the training load (ECOs), as well as the evolution of the anthropometric values and performance of three elite U23 female triathletes over a season. (2) Methods: General training data and performance data relating to the swimming, cycling, and running legs of the 2021 season were described. The training intensity distribution (TID) was presented using the triphasic model, while the training load was based on the ECO model. An anthropometric analysis was also conducted in accordance with the ISAK standards. (3) Results: Triathletes increased their VO_2max_ in cycling (6.9–10%) and running (7.1–9.1%), as well as their power and speed associated with the VO_2max_ (7.7–8.6% in cycling and 5.1–5.3% in running) and their swimming speed associated with the lactate thresholds (2.6–4.0% in LT2 and 1.2–2.5% in LT1). The triathletes completed more than 10 h of weekly average training time, with peak weeks exceeding 15 h. The average TID of the three triathletes was 82% in phase 1, 6% in phase 2, and 12% in phase 3. A decrease in the sum of skinfolds and fat mass percentage was observed during the season in the three triathletes, although the last measurement revealed a stagnation or slight rise in these parameters. (4) Conclusions: The triathletes performed a combination of two training periodization models (traditional and block periodization) with a polarized TID in most of the weeks of the season. Improvements in performance and physiological parameters were observed after the general preparatory period as well as a positive body composition evolution throughout the season, except at the end, where the last measurement revealed stagnation or a slight decline. This study can be useful as a general guide for endurance coaches to organize a training season with female U23 triathletes.

## 1. Introduction

Triathlon is a multidisciplinary endurance sport in which swimming, cycling, and running are timed consecutively without interrupting the chronometer [1]. The so-called short-distance triathlon includes sprints (750 m of swimming, 20 km of cycling, and 5 km of running) and Olympic distances (1500 m of swimming, 40 km of cycling, and 10 km of running). A major difference between short- and long-distance triathlon events (such as Ironman)—apart from distance—is that in short-distance triathlon [2], some national federations organize their national elite championships. In Spain for example, following a prior classification in a qualifying race, 100 triathletes compete in the national Sprint and Olympic championship [1]. Triathlon, like other endurance sports, such as cycling, has an under-23 (U23) category, the results in this category being a good predictor of future performance in the elite category [3,4]. Furthermore, given that peak performance is achieved in short-distance triathlon at around the age of 27 in both males and females [5,6], competing in the U23 category can motivate athletes not to abandon the sport before they have achieved their best results in the elite category [7].

Moreover, in the sports literature, there is a growing interest in descriptive studies on long-term training plans for elite endurance athletes [8,9,10,11]. Some of these published studies focus on world-class athletes. Examples include the preparation of three top-five cyclists for the Giro d’Italia [8], the training intensity distribution in world-class middle- and long-distance runners [12], and the training program of a world-class triathlete preparing for the Tokyo 2020 Olympic Games [9]. However, several studies have also been published using data from full seasons or prolonged training periods in elite junior and U23 athletes [11,13,14,15]. For example, Leo et al. [11] observed how training characteristics varied across a competitive season and how they were related to the changes in the power profiles of elite U23 cyclists. For their part, Gallo et al. [14] compared training characteristics during the competitive season between junior, U23, and elite cyclists. These data can be of great interest because they can help coaches orient the training of athletes toward high performance in the elite category. Despite the latter, as emphasized in a recent review, further research is needed on prolonged periods of training in youth categories, as well as the impact on performance [16].

Men and women present some physiological differences that can affect endurance training adaptations. These differences apply to several parameters, among which are muscle contractions. Indeed, women have been reported to be more resistant to muscle fatigue [17] or with respect to endocrine responses because a woman’s hormonal system changes depending on the menstrual cycle phase [18]. Moreover, regarding the energy system, women oxidize proportionally more fat and less carbohydrates and protein than men [19]. Lastly, with respect to muscle strength, women present less absolute strength values than men and a greater gender difference has been observed in upper body strength [20].

Despite these differences, few long-term training reports have been elaborated for female athletes, leading coaches to use male athlete training data as a reference, adapting them to female athletes. Regarding endurance sports, Van Erp et al. [21] described training differences between male and female professional road cyclists, concluding that women’s training was of greater intensity than men’s but lower in volume. Tjelta et al. [22] described the career of a female marathon runner who won the New York Marathon nine times, focusing on the training volume and intensity during two of her most successful seasons. For her part, Solli et al. [23] investigated the training characteristics of a successful female world-class cross-country skier who won six gold medals at the Winter Olympic Games. Specifically, regarding the triathlon sport, Mujika [24] is the only author to have reported some training data on female athletes, providing the training characteristics of an international elite triathlete the year before the London Olympic Games of 2012. To the best of our knowledge, however, no study has hitherto been published offering short- or long-term data on training load and performance in U23 triathletes.

Finally, several training load quantification methods have been used in sports science research, such as those based on heart rate [25]; the rate of perceived effort (RPE) [26]; or heart rate variability (HRV) [27]. However, objective and subjective load equivalents (ECOs in Spanish, i.e., “equivalentes de carga objetiva y subjetiva”) seem to be the most adequate method to quantify triathlon training load, since ECOs consider the multimodal component of the triathlon sport [28,29].

Based on all the above, the aim of this study was to present the training load (ECOs), as well as the evolution of the anthropometric values and performance of three elite U23 female triathletes over a season. Our initial hypothesis was that triathletes would increase their performance in the endurance markers evaluated and decrease their sum of skinfolds throughout the season.

## 2. Materials and Methods

### 2.1. Experimental Design

A longitudinal and descriptive case study was carried out. Three female elite U23 triathletes participated in this study. The triathletes trained with the same coaches (R.C. and S.S.) and in the same training group during the season. The data presented in this study correspond to the 2021 season, in which the triathletes won the U23 national sprint triathlon team championship.

**Figure 1:**
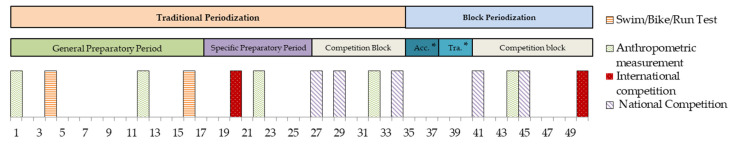
Timeline of this study. * Acc. = accumulation; Tra. = transmutation.

Training load data were collected over the course of an entire season. Physiological tests were performed at the beginning and at the end of the general preparatory period to evaluate changes in performance in swimming, cycling, and running. The test took place in weeks 4 and 16 of the season. Triathlete A performed the second test for cycling and running in week 19 due to an injury. Anthropometric measurements were also carried out throughout the season to analyze changes in the participants’ body compositions. Anthropometric measurements were taken in weeks 1, 12, 22, 33, and 44 of the season. All procedures used were approved by the Alicante University Ethics Committee (UA-2017-04-11 expedient). The triathletes gave their consent for their data to be published in this study. Figure 1 shows the timeline of this study, where the weeks of the season with the test and anthropometric values as well as an overview of the training periodization and the competitions are presented.

### 2.2. Participants

The present case report focuses on three elite U23 female triathletes who were born in 2000. Their level could be described as “highly trained/national” (tier 3) and “elite athletes/international” (tier 4) according to McKay’s framework for sports science research [30]. Alternatively, their level could be classified between “performance level 4” (highly trained) and “performance level 5” (elite/professional) according to Decroix’s guidelines to classify female subject groups in sports science research [31]. Their triathlon competing experience was over 7 years. In addition, they belonged to the regional and national performance development programs for young triathletes.

### 2.3. Data Collection

Running and cycling training zones were determined after performing until volitional exhaustion with an incremental test using a portable gas-exchange analyzer (Cosmed^®^ K4 2, Rome, Italy). A ramp protocol was used to cycle on a roller (Wahoo^®^, Atlanta, GA, USA) starting at 100 watts (W) and increasing by 5 W every 12 s [32]. Triathletes used their own bikes, and the watts were increased by a coach using a specific app for the roller. The running test was performed on a 400 m homologated track. Triathletes started at 12.1 km/h and increased by 0.3 km/h every 200 m [33]. Training intensity zones were calculated based on ventilatory thresholds (VTs) and maximum oxygen consumption (VO_2max_). The Davis criteria were used to establish these physiological markers [34]. The following variables were measured during the test: oxygen uptake (VO_2_); pulmonary ventilation (VE); ventilatory equivalent for oxygen (VE/VO_2_); ventilatory equivalent for carbon dioxide (VE/VCO_2_); and end-tidal partial pressure of oxygen (P_ET_O_2_) as well as carbon dioxide (P_ET_CO_2_). The VO_2max_ recorded the highest VO_2_ value obtained for any continuous 1-min period. VT1 was determined based on an increase in both the VE/VO_2_ and P_ET_O_2,_ with no increase in the VE/VCO_2_, whereas VT2 was determined based on an increase in both the VE/VO_2_ and VE/VCO_2_ and a decrease in the P_ET_CO_2._ The heart rate was continuously monitored during the test using radio telemetry (Polar Electro^®^, Kempele, Finland). Swimming training zones were determined based on speeds associated with different blood lactate concentrations and/or specific times to swim a distance after an incremental swimming test (7 × 200 m every 5 min) [32,35]. These training zones were calculated using the speed at lactate thresholds as a reference. Blood samples taken from the earlobe were examined using a portable lactate analyzer (Lactate Scout^®^, EKF-Diagnostics^®^, Magderburg, Germany). The standards for identifying thresholds were set as follows: a 0.5 mMol/L rise in blood lactate for lactate threshold 1 (LT1) and rising over 1.0 mMol/L for lactate threshold 2 (LT2) [36,37].

A total of 8 training zones were used during the workouts to indicate intensity and to calculate the training load. These training zones reported both internal load (HR) and external load (speed or power) data. Moreover, an RPE scale (1–10) was related to these training zones. However, to establish the training intensity distribution (TID), three training zones were mainly used: zone 1 (at or below VT1/LT1), zone 2 (between VT1/LT1 and VT2/LT2), and zone 3 (at or beyond VT2/LT2) [38]. The polarization index was also calculated to quantify the polarization level [39]. This index summarized the nature of the TID with a single variable. For a polarization index of >2.00 a.U., the TID was defined as “polarized”, while for a polarization index of ≤2.0, the TID was defined as “non-polarized”.

Anthropometric measurements were performed following standard protocols adopted by the International Society for the Advancement of Kinanthropometry (ISAK) [40] by the same anthropometrist with an ISAK certification of level 2. The thickness of 6 skinfolds (subscapular, triceps, supraspinal, abdominal, front thigh, and medial calf) was measured using a caliper calibrated to the nearest 2 mm (Holtain^®^, Dandenong, Australia). Four girths (relaxed arm, flexed arm, thigh, and calf) were measured using a flexible anthropometric steel tape (Holtain^®^, Dandenong, Australia). The sum of skinfolds was calculated, and the muscular mass and fat mass percentage were estimated using the method of Lee et al. [41] and the equation of Withers et al., respectively [42].

### 2.4. Training Characteristics and Control of the Training Load

The ECO methodology was used to calculate the training load. In short, the ECOs were calculated by multiplying the time (minutes) that the triathlete spent in every training zone (1–8) during the workout using a score value between 1 and 50 (depending on the training zone) and a specific factor of 1.0, 0.75, or 0.5 for running, swimming, and cycling, respectively [28,29]. This methodology seemed the most appropriate for triathlon because it compares different endurance activities, considering the different degrees of muscle damage, energy cost, effort densities, as well as differences in the ability to maintain a technique in the three segments [28]. The triathletes filled out a detailed training log with the information recorded in their training devices (GPS, an HR monitor, and a power meter). Subsequently, a specific software (Allinyourmind Training System1.0^®^) was used to calculate the ECOs. Furthermore, most of the training workouts were supervised and guided by the coaches.

The heart rate and rate of perceived effort (RPE) were used mainly for low-intensity workouts (zones 1 and 2). Speed and power were used to control moderate- and high-intensity workouts (zones 3–8) in running and cycling, respectively. The average pace for the 100 m was used to control moderate- and high-intensity workouts based on the training intensity zones obtained in the incremental swimming lactate test.

The triathletes also performed strength training throughout this season. As a rule, two weekly strength training sessions were performed for a large part of the season. Multi-joint exercises, both in the upper and lower body (clean, clean and press, pull up, bench press, hip thrust, squat, and Bulgarian squat) were performed by the triathletes. Three exercises per session with two or three sets ranging from five to ten repetitions were performed with “moderate effort”, always training far from muscle failure [43,44]. Once the exercise technique was mastered, the triathletes were encouraged to move the load as quickly as possible. In addition to these strength sessions, triathletes performed complementary “core” exercises in the warm-ups and cool-downs of most sessions, as well as scapular, hip, and ankle mobility exercises. These sessions and exercises were not included in the training load and were not considered in the training intensity distribution either.

### 2.5. Data Analysis

A descriptive analysis was conducted using the means and standard deviation of the variables. The percentage change was calculated to measure the differences between the tests. The analyses were carried out using a Microsoft Office Excel 2016 spreadsheet. The figures were elaborated by means of the GraphPad Prism 8.0.1 program.

## 3. Results

Table 1 shows the data of physiological and performance parameters in the two tests performed by the triathletes across the three legs in the season. The triathletes increased their VO_2max_ by between 7% to 10% in cycling and by around 5% in running, reaching values above 60 mL/kg/min in both legs. The performance improvement can be observed in all the markers shown in the table in terms of speed at lactate thresholds in swimming or power and speed at ventilatory thresholds in cycling and running, respectively. An increase in average speed can also be observed in the last swimming test repetition, as well as in the power and speed associated with the VO_2max_.

**Figure 2:**
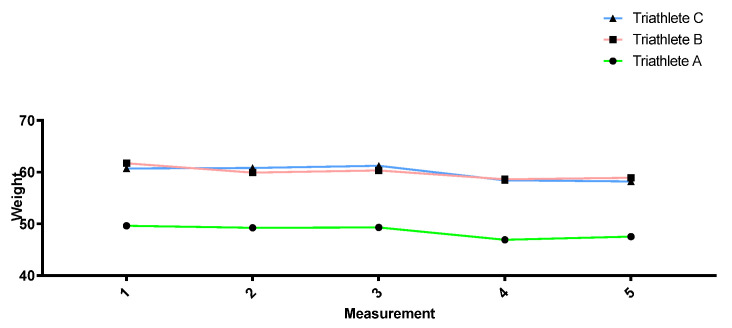
Weight evolution throughout the season.

**Figure 3:**
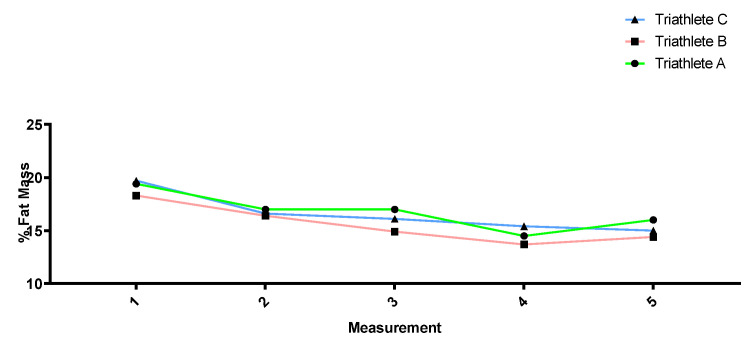
Percentage of fat mass evolution throughout the season.

**Figure 4:**
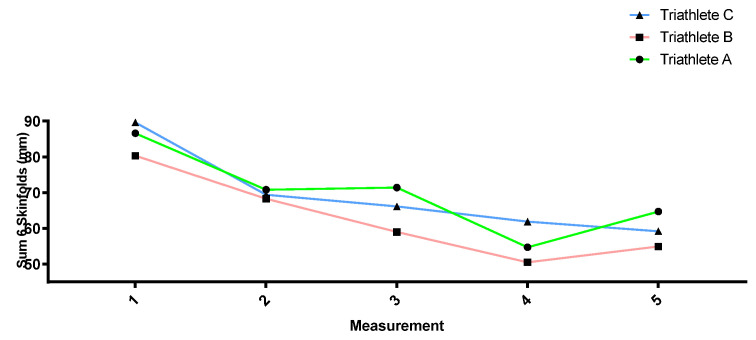
Sum of skinfolds (mm) evolution throughout the season.

**Figure 5:**
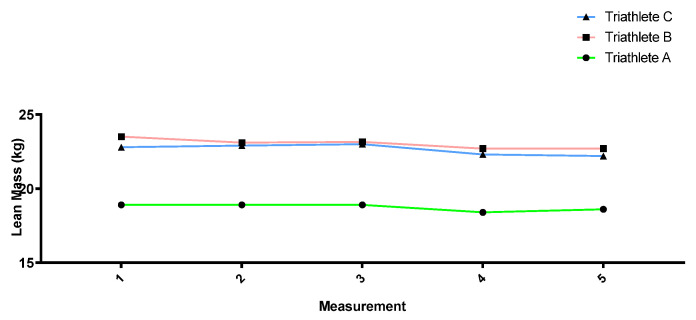
Lean mass (kg) evolution throughout the season.

Figure 2, Figure 3, Figure 4 and Figure 5 show how the anthropometric parameters evolved throughout the season. Almost no changes in weight (kg) or lean mass (kg) were observed throughout the season in the three triathletes. However, the sum of skinfolds (mm) as well as the fat percentage did progressively decrease from measurement 1 to measurement 4, except in triathlete A, for whom no decrease was found between measurements 2 and 3. All three triathletes showed a stagnation and even a slight increase in these parameters between measurements 4 and 5.

Table 2 summarizes the 2021 season training characteristics of the three triathletes. Triathlete A completed a higher average of weekly training hours as well as a greater weekly average training load (ECOs) than triathletes B and C. Although the three triathletes’ minimum weekly ECO load was highly similar, triathlete A completed almost 200 ECOs more than triathlete C in the season’s week of maximum training. The distribution of ECOs by segment was equal for swimming and running, and lower for cycling. Triathletes B and C presented an average polarized training intensity distribution, while triathlete A showed a non-polarized average because the polarization index value did not reach 2.0 a.U. However, the training load distribution showed how the triathletes performed more than 50% of it in zone 3. Regarding the competitions, most of the triathletes’ races were national and local, but they also took part in at least one elite European triathlon cup.

**Figure 6:**
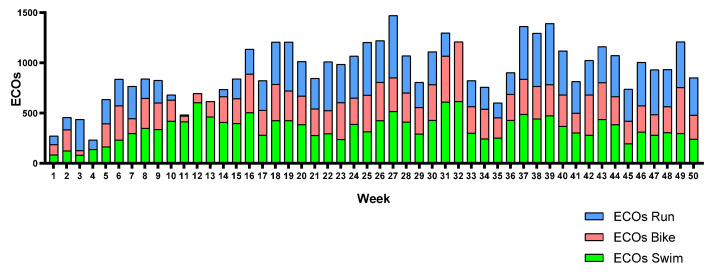
Triathlete A weekly training load by leg.

**Figure 7:**
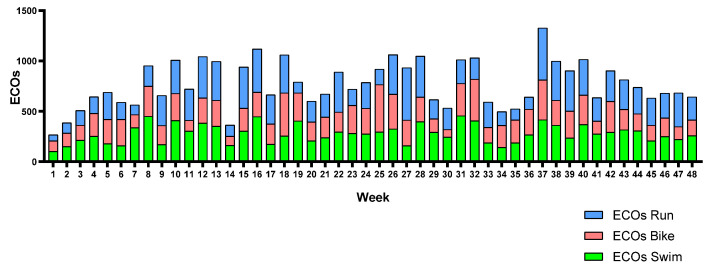
Triathlete B weekly training load by leg.

**Figure 8:**
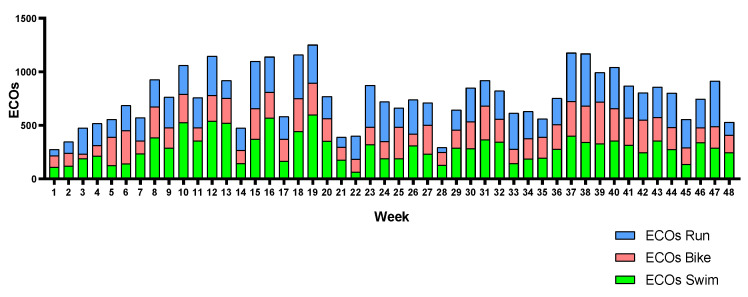
Triathlete C weekly training load by leg.

Figure 6, Figure 7 and Figure 8 show each triathlete’s weekly training load during the season. The training load can be observed to be equally distributed across the three legs in almost all weeks. Triathlete A suffered a knee injury and had to reduce her running training load from weeks 10 to 14.

**Figure 9:**
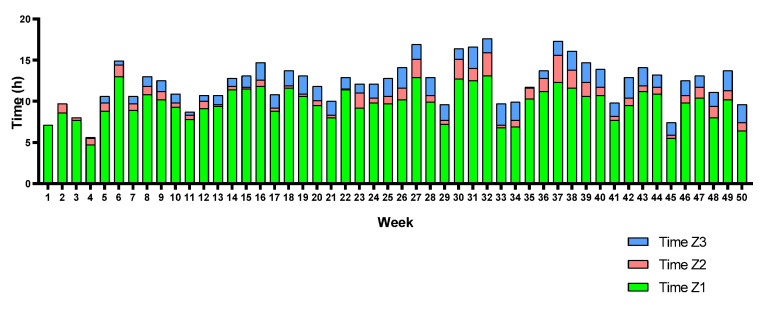
Triathlete A weekly training volume and TID.

**Figure 10:**
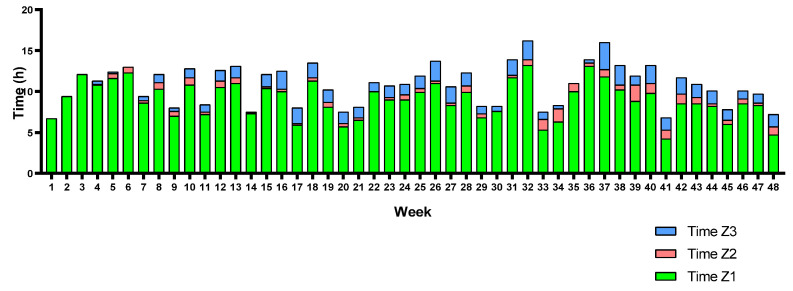
Triathlete B weekly training volume and TID.

**Figure 11:**
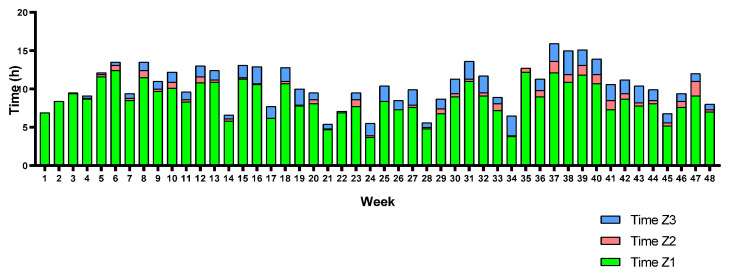
Triathlete C weekly training volume and TID.

Moreover, each triathlete’s training volume (weekly hours) and TID are represented in Figure 9, Figure 10 and Figure 11. Triathletes B and C presented a polarized TID model in most weeks of the season, while triathlete A accumulated a greater training volume percentage in Z2 than the other triathletes. Despite this, although the seasonal TID average does not indicate a “polarized” polarization index value for triathlete A, a polarized TID can, in fact, be observed in most weeks of the season. There is a large training volume in Z1 and a higher percentage in Z3 than in Z2.

Two major competition blocks were performed by the triathletes in the season. In the first, from weeks 27 to 34, the qualifiers raced for the elite Spanish Championship sprint and Olympic distance. In the second block, from week 41 to the end of the season, the national elite championships (sprint and Olympic distance), the relay elite Spanish championship, and the Spanish university championship took place. The triathletes followed a traditional periodization to prepare for the first block of competitions, where the general preparatory period lasted from weeks 1 to 16 and the specific preparatory period from weeks 17 to 26. The triathletes competed in one international race and other regional races during this specific preparatory period. Triathlete B also competed in the Spanish national duathlon championship as part of her preparation. Moreover, the triathletes followed a block periodization to prepare the second competition block with an accumulation block from weeks 35 to 37 and a transformation block from weeks 38 to 40. The accumulation was characterized by progressively increasing the training volume to achieve the training volume peak of the season in the last week 37. During the transformation block, the triathletes performed higher-intensity training sessions but reduced the volume slightly. Thus, a higher training load accumulation was observed from weeks 37 to 39. The training load started to decrease in week 40 in order to favor adaptations and increase performance in the main competitions of the season.

The main results of the triathletes in the season include triathlete A’s victory at the university national championship and coming first place for teams in the national U23 Spanish championship sprint distance, where triathlete C finished second, triathlete A fourth, and triathlete B seventh. Moreover, they came fifth in the Spanish time-trial triathlon championship for elite teams and in the elite Spanish triathlon relay championship.

## 4. Discussion

The present study described the training characteristics and how the physiological performance and anthropometric markers of three U23 female triathletes evolved throughout a season. Previous research has reported the relevance of junior and U23 category performance, owing to a strong correlation with future elite category performance in endurance sports, such as cycling [3]. In line with this, the triathletes presented significant achievements during the season, persistently occupying leading positions in elite national competitions in this category.

The triathletes showed 6.9% to 10% increases in VO_2max_ values in cycling and running, achieving high VO_2max_ values, in line with the other VO_2max_ values in women elite endurance athletes such as cyclists [45] or runners [46], though higher than those of rowers [47] or cross-country skiers [48]. Nevertheless, these values were slightly lower than those reported for other world-class endurance athletes [23,49,50]. The differences can be explained not only by performance levels but also by age differences and the total number of years of systematic training [23]. In relation to performance, the triathletes also showed power and speed values associated with the VO_2max_ and ventilatory thresholds that were in line with or slightly lower than those reported for elite cyclists and runners [49,51]. In this respect, other aspects such as energy cost or the so-called “durability”—i.e., the magnitude of deterioration of physiological profiling characteristics over time during prolonged exercise—mark the differences between triathlete and specialist performance in each modality [52].

The triathletes performed over 10 h of weekly average training volume, several weeks including more than 15 h of training. This training volume is in line with other training volumes reported for elite female athletes, such as track and field athletes [53], or long-distance runners [49,54], but less than the training volume reported for elite female cross-country skiers who performed an average of over fifteen weekly hours [55]. The triathletes trained considerably less than an Olympic female triathlete reported in a case study (10.4–12.2) [24]. The latter, however, was a professional triathlete who trained for many years preparing for the Olympic Games, where she obtained an Olympic diploma. The difference is therefore justified [24].

Triathletes B and C followed an average polarized TID (polarized index of >2.0 a.U). Triathlete A, however, although also close to that value, had an average TID that could not be defined as polarized. Yet, similar TIDs were not observed every week because some weeks, the distribution was more pyramidal with a greater amount of training time at moderate intensity (phase 2). Both polarized and pyramidal TIDs have been described as appropriate ways to distribute endurance athlete training volume [56,57,58,59]. In addition, the periodization evolution from a pyramidal TID to a polarized TID has been shown to produce greater performance effects than other TID combinations in well-trained runners [60]. This evolution was visible in triathletes when they prepared the second block of competitions in the second part of the season. Both models (pyramidal and polarized) presented a high training volume percentage (around 80%) at low intensity (phase 1). This trend was observed for the three triathletes across almost all the weeks. A large training volume at low intensity leads to skeletal muscle adaptations owing to an increase in the respiratory capacity and mitochondrial content of muscle fibers [61]. Long-duration/low-intensity training sessions also contribute the ability of the athlete to recover from high-intensity efforts as well as to maintain relatively high muscular power outputs for long durations [62]. Even though a polarized or pyramidal TID model was clearly followed in almost all weeks, the training load distribution was composed by around 50% of the ECOs in zone 1 and the other 50% in zones 2 and 3, following the called “50/50 rule” [4,9].

The triathletes followed two different periodization models during the season. They followed a traditional periodization during the first part of the season, because they had a large number of weeks until they reached the first block of competitions. In line with this, a long (general and specific) preparatory period was possible, where the training volume was progressively increased in the first part of the preparation, and the higher-intensity workouts were increased from weeks 8 to 9 of preparation onward. The traditional periodization model can be useful with young endurance athletes who do not have multi-peak performances due to an excessive number of competitions. They can benefit from a “mixed” training program, developing several capacities and abilities at the same time, while remembering the accumulated amount of fatigue and the negative interactions that can occur between their capacities [63]. However, a block periodization (ATR) was followed by the triathletes to prepare for the second block of competitions. As the triathletes had less preparation time for this second block, they had to intensify their training: they focused on the training volume with a large number of long workouts at low or moderate intensity during the first weeks in the accumulation block and with more specific intensity training sessions in the transmutation block. Block periodization has been described as an alternative to traditional periodization for experienced elite athletes who must achieve multiple performance peaks due to a large number of competitions during the season [63]. Although the effectiveness of block periodization has been demonstrated for world-class and elite endurance athletes [4,64,65], no consensus has been reached regarding its superiority with respect to traditional periodization [66,67].

The combination of the two periodization models as presented in this study can be of interest in the case of seasons with two competitive blocks, but that are not evenly distributed in temporal terms. Indeed, the number of weeks to prepare for the first block of competitions is considerably greater than for the second, as in the case of these three triathletes.

In relation to the anthropometric measurements taken at different times in the season, triathlete weight remained relatively stable throughout the season, but a decrease in the sum of skinfolds could be observed together with a reduction in the fat mass percentage. This change can be considered positive since body composition has been related to sports performance, with a low percentage of body fat being common in elite endurance athletes [68,69,70]. However, the athletes’ last measurement showed a stabilization or slight increase in these parameters. The reason may be that the last measurement was performed during the second competition period when the triathletes could not accumulate a heavy training load owing to an excessive number of competitions. Moreover, poorer diets during competition trips may also explain the slight increase in the sum of skinfolds and the fat mass percentage at the end of the season.

The triathletes also performed strength training sessions throughout the season. An adequate structure of concurrent training can be beneficial in endurance sports, mainly because it improves the energy cost of locomotion [71]. In this respect, a “low–moderate effort” in strength training, with repetitions, and keeping away from muscle failure could favor positive performance adaptations while preventing negative effects such as muscle hypertrophy or excessive fatigue that could affect endurance workouts [72].

Regardless of the possible improvements that strength training can bring to sports performance, its implementation could also have a significant protective effect against injuries [73]. Thus, considering the greater prevalence of overuse injuries in female athletes [74], it seems rather important to incorporate strength training into triathlete training programs.

Finally, this study presents certain limitations, and the results must be interpreted with caution. We described the training process of three female elite U23 triathletes and the data can be useful for coaches. However, the context in which the participants developed their performance (training place, training partners, and group of coaches) makes the process impossible to repeat exactly for other endurance athletes. Moreover, these training characteristics cannot be extrapolated to other populations, such as recreational athletes. In addition, a lack of studies on the detailed training characteristics of young endurance female athletes makes it difficult to compare our results. Furthermore, it would have been interesting to repeat a greater number of physiological measurements throughout the season, which was not possible because the training and competition dynamics did not allow us to schedule other test weeks. Finally, it is important to remark that there is no single path to follow when planning training. We do not know, therefore, whether these triathletes would have achieved better results with a different training program.

## 5. Conclusions

The present study detailed the training characteristics of three elite U23 female triathletes as well as the evolution of their body compositions and performance throughout a season. The triathletes improved their VO_2max_ in cycling and running by 8.3% and 7.7%, respectively, as well as the power and speed associated with the VO_2max_ in cycling and running by 8.2% and 5.7%, respectively. Power and speed associated with ventilatory or lactate thresholds also improved in the three segments after the general preparatory period. The triathletes saw their sum of skinfolds and fat mass percentage decrease during the season, except in the last measurement, which revealed stagnating or slightly higher values. The triathletes mainly performed a polarized TID during most of the various season weeks, and a combination of two periodization models (traditional and ATR) was applied. The above data can be of interest to coaches who can use them as a basis to structure the training of young elite female triathletes during a season. The training volume, the TID proposed, and the combination of the two periodization models have been observed to have positive effects on performance, causing athletes to obtain good results during the season without major injuries. Therefore, this training plan can be used as a general guide for triathlon coaches, who must adapt this information to their context and the level of their athletes. Future studies should try to expand the sample of participants in this research, comparing the effects of different training methodologies and even trying to observe the evolution of performance in the longer term, not just during one season.

## Figures and Tables

**Table 1:** Triathlete physiological and performance markers.

	Triathlete A			Triathlete B			Triathlete C		
	Test 1	Test 2	%Change	Test 1	Test 2	%Change	Test 1	Test 2	%Change
**Swim**									
S_L200_ (m/s)	1.37	1.39	1.4	1.35	1.39	2.8	1.41	1.43	1.4
LT2	1.28	1.32	2.6	1.25	1.30	3.9	1.28	1.33	4.0
LT1	1.22	1.25	2.5	1.16	1.19	2.4	1.22	1.23	1.2
HRMax (bpm)	184	184	0.0	184	187	1.6	184	186	1.1
**Bike**									
VO_2Max_ (mL/kg/min)	59.8	63.9	6.9	60.7	66.8	10	61.3	66.2	8.0
W VO_2Max_	260	280	7.7	290	315	8.6	290	315	8.6
W VT2	200	215	7.5	240	260	8.3	230	250	8.7
W VT1	155	170	9.7	175	195	11.4	160	190	18.8
W/kg VO_2Max_	5.3	5.9	12.5	4.7	5.3	12.2	4.8	5.2	8.4
W/Kg VT2	4.1	4.6	12.3	3.9	4.3	12.0	3.8	4.1	8.5
W/kg VT1	3.1	3.6	14.6	2.8	3.3	15.0	2.6	3.1	18.6
HRMax (bpm)	181	182	0.6	187	187	0.0	183	182	−0.5
**Run**									
VO_2Max_ (mL/kg/min)	60.3	64.6	7.1	59.4	64.8	9.1	63.1	67.5	7.0
S VO_2Max_	17.2	18.1	5.2	16.9	17.8	5.3	17.5	18.4	5.1
S VT2	15.1	16.3	7.9	14.5	15.4	6.2	14.8	16.0	8.1
S VT1	13.3	13.9	4.5	13.0	13.3	2.3	13.0	13.6	4.6
HRMax (bpm)	186	188	1.1	191	195	2.1	193	197	2.1

S_L200_ = average speed in the last test repetition; LT = lactate threshold; HRMax = maximum heart rate; bpm = beats per minute; VO_2max_ = maximum oxygen uptake; W = Watts; VT = ventilatory threshold; S = speed.

**Table 2:** Summary of training characteristics of the season.

	Triathlete A	Triathlete B	Triathlete C
**Training Load**			
WA (total ECOs) ($\bar{x}$ (ds))	922 (276)	770 (226)	760 (251)
Maximum ECOs week	1361	1327	1176
Minimum ECOs week	271	266	274
%ECOs swimming ($\bar{x}$ (ds))	36.6 (13.2)	36.1 (11.9)	37.5 (16.9)
%ECOs cycling ($\bar{x}$ (ds))	29.3 (10.7)	29.5 (11.4)	27.8 (10.4)
%ECOs running ($\bar{x}$ (ds))	34.1 (14.9)	34.3 (14.9)	34.8 (13.2)
%ECOs Z1 ($\bar{x}$ (ds))	50.8 (13.9)	55.1 (17.1)	53.4 (16.8)
%ECOs Z2 ($\bar{x}$ (ds))	9.7 (6.5)	7.8 (7.7)	5.7 (4.1)
%ECOs Z3 ($\bar{x}$ (ds))	40.5 (14.5)	40.1 (13.7)	43.2 (14.4)
**Training Volume**			
Total weeks (n)	50	48	48
WA training time (h) ($\bar{x}$ (ds))	12.2 (2.7)	10.8 (2.4)	10.4 (2.7)
Maximum weekly hours	17.6	16.2	15.9
Minimum weekly hours	5.5	6.7	5.4
% Training time Z1 ($\bar{x}$ (ds))	80.2 (6.9)	83.3 (8.7)	82.7 (8.5)
% Training time Z2 ($\bar{x}$ (ds))	7.5 (4.2)	5.8 (4.6)	4.5 (3.3)
% Training time Z3 ($\bar{x}$ (ds))	12.3 (6.0)	11.9 (6.0)	12.8 (7.4)
Polarization index (a.u.)	1.92	2.08	2.13
**RACES**			
International races (n)	2	1	1
National races (n)	7	9	8
Local races (n)	5	5	4

WA = weekly average; a.u. = arbitrary units; n = number.

## Data Availability

The data is available on https://drive.google.com/drive/folders/1PRHnojvVYndEU4hRL_Oa9Cop11aA9E5H?usp=sharing, accessed on 16 January 2024.
